# Prevalence and Risk Factors of Post‐Extraction Complications in a Western Australian Tertiary Dental Hospital: A Retrospective Cross‐Sectional Study

**DOI:** 10.1111/adj.13082

**Published:** 2025-06-10

**Authors:** Peter Dignam, Mariam Elshafey, Aparna Jeganathan, Magdalen Foo, Joon Soo Park, Manorika Ratnaweera

**Affiliations:** ^1^ UWA Dental School The University of Western Australia Nedlands Western Australia Australia; ^2^ Centre for Optimisation of Medicines The University of Western Australia Crawley Western Australia Australia; ^3^ Health Equity College The University of Western Australia Crawley Western Australia Australia

**Keywords:** extraction, post‐extraction complication, post‐operative complication, prevalence, risk factors

## Abstract

**Objectives:**

Dental extractions are routine procedures, yet they carry a risk of post‐operative complications that can negatively affect patients' quality of life. This study aimed to identify patient‐, tooth‐ and clinician‐related risk factors associated with post‐extraction complications at Western Australia's only tertiary dental hospital, to inform clinical decision‐making and improve outcomes by identifying high‐risk groups.

**Methods:**

A retrospective audit of patient records was conducted using data from individuals who underwent dental extractions during the audit period. Extracted variables included demographic details, medical history (e.g., smoking status, diabetes, hypertension), extraction characteristics (e.g., simple vs. surgical, tooth location) and clinician factors (e.g., level of training, anaesthesia type). Post‐operative complications were identified through clinical follow‐up notes. Descriptive and inferential statistical analyses were used to determine significant associations.

**Results:**

The overall post‐extraction complication rate was 11.0%. The most common complications were persistent post‐operative pain (4.1%) and alveolar osteitis (3.4%). Statistically significant patient‐related risk factors included smoking, uncontrolled diabetes and hypertension. Surgical extractions and mandibular tooth removals were associated with higher complication risks. Extractions performed by students and those conducted under general anaesthesia were also linked to increased complications.

**Conclusions:**

Approximately 1 in 10 patients experienced post‐operative complications. Identifying modifiable risk factors and minimising surgical complexity may reduce adverse outcomes. Operator experience and anaesthesia choice should be considered during preoperative planning.

**Summary**

- Clinical Relevance:
  ○ This study highlights the importance of structured preoperative risk assessment that integrates medical comorbidities, extraction complexity and operator training level. Tailoring surgical planning and post‐operative monitoring based on individual risk profiles may improve clinical outcomes, reduce avoidable complications and support safer, more effective delivery of care in tertiary and teaching settings.

## 1 Introduction

Dental extractions are a common procedure performed in many dental practices worldwide. The latest report from the Australian Institute of Health and Welfare estimated that 13.5% of the 2010 Australian population previously had a dental extraction [1]. They reported that the most common indication for those extractions was tooth decay, at 38.6%, followed by wisdom tooth impaction, at 30.9%, then trauma, at 26.4% and finally periodontal disease, at 9.5%.

Although extractions are generally considered safe procedures, it is possible for complications to arise following them. Possible complications include alveolar osteitis, osteomyelitis, abscess, facial cellulitis, long‐standing pain, haemorrhage, haematoma, trismus, oedema, inferior alveolar nerve damage, lingual nerve damage, mental nerve damage, osteonecrosis of the jaw, osteoradionecrosis of the jaw and oral antral communication [2]. Such complications may result in an increased need for post‐operative visits and decreased quality of life. Therefore, preventing these complications can assist in decreasing post‐operative morbidity and alleviating the economic burden of disease in the form of lost time from work and healthcare expenses.

Specific patient‐related, tooth‐related and clinician‐related factors may significantly influence the likelihood of a post‐extraction complication. Therefore, the identification of these risk factors is important to enable better recognition of high‐risk patients. Modifiable risk factors can also be targeted to reduce the patient's overall risk level. Instances of patient‐related risk factors that have been established in the literature include older age [3], female sex [4], some systemic health conditions [5, 6, 7, 8], some medications [9], tobacco smoking [10, 11, 12] and poor oral hygiene levels [13, 14, 15, 16]. Meanwhile, the identified tooth‐related risk factors include complex extractions [17], mandibular extractions [18, 19], third molar extractions [20, 21] and teeth that were indicated for extraction due to either impaction or the presence of pericoronitis [22, 23]. Finally, the clinician‐related risk factors include lower levels of experience [24], use of general anaesthesia over local anaesthesia [25, 26], use of envelope flaps over triangular flaps [14, 27, 28] and the types of pre‐/post‐operative instructions given [29, 30, 31, 32, 33].

Without a clear understanding of the prevalence of complications following tooth extraction, it is difficult to determine whether current measures can prevent these complications. Thus, this research project aims to identify the prevalence of post‐extraction complications at Western Australia's tertiary dental hospital and determine which population groups are at the highest risk of post‐extraction complications. Identifying the factors that either increase or decrease the risk of post‐extraction complications may provide insight into the appropriateness of the current dental extraction protocols.

## 2 Materials and Methods

### 2.1 Study Design

A retrospective epidemiological cross‐sectional study design was conducted in accordance with the Strengthening the Reporting of Observational Studies in Epidemiology (STROBE) statement guidelines [34].

### 2.2 Setting and Participants

The study population comprised patients who underwent one or more dental extractions at the Oral Health Centre of Western Australia (OHCWA) between January 2017 and December 2022 inclusive. Inclusion criteria specified patients aged 12 years or older at treatment. Individuals younger than 12 years were excluded due to differences in physiological healing processes between children and adults, which may have confounded the results [35, 36]. Additional exclusion criteria included records with purged clinical notes in the Titanium Oral Health Management (TOHM) system (Titanium Solutions, Auckland), as these could not be comprehensively analysed. Cases were also excluded if the patient's medical history had not been updated on the day of extraction, if the indication for extraction was not documented or if the specific type of post‐operative complication was not recorded. These omissions indicated substandard medical record‐keeping and could compromise data integrity [37].

Data were retrieved from OHCWA's TOHM system. Medical records that met the inclusion and exclusion criteria were de‐identified, extracted and aggregated. The dataset was then securely stored before analysis. To identify eligible cases, a surrogate marker search strategy was employed within the Titanium database to isolate relevant entries associated with post‐extraction complications.

### 2.3 Sample Size

Between January 2017 and December 2022, 19,355 tooth extractions were performed at OHCWA. Using a 95% confidence level and a 5% margin of error, the minimum required sample size was calculated to be 377 extractions. The final sample included 1143 patients who underwent 2343 extractions, exceeding the minimum required to ensure adequate statistical power.

### 2.4 Data Extraction

The data were categorised based on specific patient‐related, tooth‐related and clinician‐related variables. The complete list of subcategories within each of the listed variables is outlined in Appendix S1. To determine if a post‐extraction complication had occurred, the clinical notes from the subsequent appointments were screened until healing of the extraction site was complete or no further clinical notes were available. Unless a specific post‐operative complication was diagnosed, the dataset was excluded from the study. Such complications included alveolar osteitis, osteomyelitis, abscess, facial cellulitis, long‐standing pain, nerve impairment, haemorrhage, haematoma, trismus, osteonecrosis of the jaw and oral antral communication. Agreed upon definitions were used in cases where the clinician identified but did not diagnose the post‐operative complication (Appendix S2). Furthermore, to ensure inter‐rater reliability, the three data collectors reviewed and agreed upon the entries of eight cases during each of the 6 years. This consisted of reviewing the first two cases, the last two cases and four random cases each year.

### 2.5 Statistical Analysis

The data were imported as a comma‐separated values (CSV) file. The quantitative data were then analysed using the dependent variable (post‐operative complications) and the independent variables (patient‐related, tooth‐related and clinician‐related factors). The data were analysed using descriptive statistic odds ratio for each category per adverse outcome. SPSS version 29.04 (IBM Corp., Armonk, NY) and Jupyter Notebook 6.4.12 (Jupyter URL: http://jupyter.org/) were used. Descriptive statistics were calculated for each variable to summarise the demographic and summary characteristics of the study population. After the time trend was generated, Pearson's *r* (range = −1 to 1) and a two‐tailed *p* value were generated to assess the correlation [38]. Furthermore, logistic regression was employed to estimate odds ratios (ORs) for specific predictors, including health conditions. The statistical significance was set at *p* < 0.05.

## 3 Results

### 3.1 Participants

Between January 2017 and December 2022, a total of 19,355 extractions were recorded. Following surrogate marker identification and manual screening, 3422 extractions were examined for eligibility. Of these, 2343 extractions across 1143 patients met inclusion criteria and were included in the analysis. A total of 1079 extractions were excluded due to incomplete or purged records, including missing updated medical histories (*n* = 111), absent indication for extraction (*n* = 154), purged clinical notes (*n* = 817) or unspecified complications (*n* = 2). In 15 cases, both the medical history and indication were missing.

### 3.2 Patient‐Related Factors

The mean age at extraction was 48.9 years, with 30.2% (*n* = 707) of extractions performed in patients aged ≥ 65 years. Female patients accounted for 51.9% (*n* = 1216), males for 47.8% (*n* = 1119) and 0.3% (*n* = 8) identified as another gender. Medication use was prevalent in 61.0% (*n* = 1429) of extractions. Notably, 15.9% (*n* = 372) involved patients on anticoagulants or antiplatelets, 6.5% (*n* = 153) involved immunosuppressant use and 2.0% (*n* = 47) involved synthetic oestrogens or progestins. Smoking history was reported in 37.0% of cases, including 23.6% of current smokers and 13.3% of former smokers. In terms of medical comorbidities, 29.9% (*n* = 700) of extractions were in patients with hypertension, 11.8% (*n* = 276) in those with diabetes mellitus and 22.3% (*n* = 523) in individuals reporting depression or anxiety. Other conditions of note included respiratory diseases (18.5%, *n* = 433), cardiovascular disease (13.0%, *n* = 304), bleeding disorders (7.3%, *n* = 171) and history of radiotherapy or chemotherapy (8.9%, *n* = 209).

### 3.3 Tooth‐Related Factors

The most common extraction indication was a hopeless restorative prognosis (43.7%, *n* = 1023), followed by impaction (21.7%, *n* = 508), elective extraction (11.4%, *n* = 266) and hopeless periodontal prognosis (9.2%, *n* = 215). The distribution was nearly equal between mandibular (50.9%, *n* = 1193) and maxillary (49.1%, *n* = 1150) teeth. Mandibular third molars (teeth 38 and 48) were the most frequently extracted, followed by maxillary third molars (teeth 18 and 28) (Table 1).

**TABLE 1:** Frequency of tooth extraction divided per tooth and quadrant.

	Quadrant 1	Quadrant 2	Quadrant 3	Quadrant 4	Deciduous
Tooth 48				11.69%	
Tooth 38			11.22%		
Tooth 18	9.22%				
Tooth 28		8.41%			
Tooth 46				3.33%	
Tooth 26		3.07%			
Tooth 37			2.90%		
Tooth 47				2.90%	
Tooth 36			2.86%		
Tooth 24		2.77%			
Tooth 14	2.56%				
Tooth 27		2.52%			
Tooth 17	2.43%				
Tooth 25		2.35%			
Tooth 13	2.30%				
Tooth 16	2.30%				
Tooth 35			2.26%		
Tooth 15	2.05%				
Tooth 23		2.01%			
Tooth 22		1.84%			
Tooth 11	1.79%				
Tooth 32			1.75%		
Tooth 45				1.75%	
Tooth 44				1.71%	
Tooth 21		1.66%			
Tooth 12	1.62%				
Tooth 31			1.58%		
Tooth 34			1.45%		
Tooth 41				1.45%	
Tooth 33			1.41%		
Tooth 42				1.41%	
Tooth 43				1.07%	
Tooth 75					0.13%
Tooth 65					0.09%
Tooth 53					0.04%
Tooth 55					0.04%
Tooth 85					0.04%
Total	24.27%	24.63%	25.43%	25.31%	0.34%

### 3.4 Clinician‐Related Factors

The operator was a student in 36.2% (*n* = 847) of cases, a general dentist in 38% (*n* = 901) of cases and an oral surgeon or oral surgery resident in 25.4% (*n* = 595) of cases. Most teeth, 71.9% (*n* = 1684), were extracted under local anaesthesia. Mouth rinse was given alongside 12.5% (*n* = 292) of tooth extractions, antibiotics were prescribed in 6.4% (*n* = 154) of cases and intra‐socket gel foam was placed immediately following the extraction in 14.1% (*n* = 331) of extraction sites.

### 3.5 Post‐Operative Complications

A total of 258 extractions (11.0%) were associated with documented post‐operative complications. The most common were long‐standing pain (4.1%, *n* = 96), alveolar osteitis (3.4%, *n* = 79), trismus (1.4%, *n* = 33) and nerve impairment (1.4%, *n* = 32). Other complications included oro‐antral communication (0.5%), haemorrhage (0.4%), osteonecrosis of the jaw (0.3%), abscess formation (0.3%), haematoma (0.3%) and facial cellulitis (0.2%). Among patients with nerve impairment, 46.9% had resolved symptoms, 50% remained unresolved at the last follow‐up and 3.1% were lost to follow‐up.

### 3.6 Annual Trends

Year‐by‐year analysis showed no statistically significant variation in complication rates over the 6‐year audit period (*p* > 0.05).

### 3.7 Factors Associated With Post‐Extraction Complications

Logistic regression analysis identified several statistically significant associations (Table 2). Smoking and uncontrolled diabetes were strongly associated with an increased risk of alveolar osteitis (OR 2.59 and 7.41, respectively; *p* < 0.005). Surgical extractions with bone removal significantly increased the likelihood of multiple complications, including nerve impairment (OR 26.77), trismus (OR 4.47) and haematoma (OR 18.28).

**TABLE 2:** Predisposing factors to post‐operative complications.

	OR	95% CI	*p*
**Alveolar osteitis**			
Smoker	2.591	1.640, 4.094	0.000
Smoker: current	2.534	1.606, 3.998	0.000
Diabetes: uncontrolled	7.408	2.048, 26.794	0.002
Any medication	1.684	1.023, 2.775	0.041
Indication: unrestorable	2.288	1.437, 3.642	0.000
Indication: impaction	0.398	0.190, 0.831	0.014
Arch: maxillary	0.625	0.394, 0.992	0.046
Arch: mandibular	1.599	1.008, 2.538	0.046
Clinician: student	2.287	1.455, 3.595	0.000
Clinician: general dentist	0.365	0.206, 0.644	0.001
Anaesthesia: LA	2.234	1.200, 4.157	0.011
Anaesthesia: GA	0.405	0.213, 0.771	0.006
**Abscess**			
Diabetes: uncontrolled	23.684	2.731, 205.397	0.004
Clinician: student	5.329	1.073, 26.463	0.041
Rinse given	4.265	1.014, 17.939	0.048
**Facial cellulitis**			
Indication: impaction	14.556	1.623, 130.516	0.017
**Pain**			
Blood pressure: normal	0.578	0.383, 0.872	0.009
Blood pressure: high	1.963	1.300, 2.966	0.001
Indication: impaction	1.598	1.022, 2.499	0.040
Indication: pericoronitis	2.488	1.323, 4.680	0.005
Surgical: with bone removal	1.687	1.083, 2.627	0.021
**Nerve impairment**			
Indication: impaction	4.201	2.083, 8.472	0.000
Indication: other	2.908	1.242, 6.805	0.014
Non‐surgical: simple	0.027	0.004, 0.200	0.000
Surgical: with bone removal	26.772	9.346, 76.695	0.000
Arch: maxillary	0.033	0.004, 0.239	0.001
Arch: mandibular	30.653	4.178, 224.917	0.001
Clinician: student	0.180	0.055,0.592	0.005
Anaesthesia: LA	0.106	0.046,0.246	0.000
Anaesthesia: GA	9.488	4.083, 22.046	0.000
Antibiotics given	5.933	2.695,13.053	0.000
Antibiotics given: after	4.574	1.845, 11.339	0.001
**Haemorrhage**			
Smoker: current	4.064	1.088, 15.188	0.037
Rinse given	14.379	3.576, 57.812	0.000
Rinse given: before	10.014	2.672, 37.530	0.001
**Haematoma**			
Epilepsy	62.162	12.144, 318.199	0.000
Indication: impaction	18.231	2.125, 156.397	0.008
Surgical: with bone removal	18.277	2.130, 156.795	0.008
Anaesthesia: LA	0.078	0.009, 0.666	0.020
Anaesthesia: GA	12.922	1.507, 110.814	0.020
**Trismus**			
Allergy	2.854	1.421, 5.734	0.003
Age: 65+ years old	0.228	0.069, 0.750	0.015
Sex: male	0.107	0.033, 0.351	0.000
Sex: female	9.477	2.884, 31.141	0.000
Indication: unrestorable	0.175	0.061, 0.499	0.001
Indication: impaction	11.821	5.298, 26.371	0.000
Non‐surgical: simple	0.117	0.041, 0.333	0.000
Surgical: with bone removal	4.469	2.236, 8.931	0.000
Arch: maxillary	0.446	0.211, 0.942	0.034
Arch: mandibular	2.241	1.062, 4.729	0.034
Clinician: student	0.112	0.027, 0.469	0.003
Clinician: oral surgeon or oral surgery registrar	3.184	1.598, 6.342	0.001
Anaesthesia: LA	0.165	0.078, 0.349	0.000
Anaesthesia: GA	6.080	2.878, 12.846	0.000
**Osteonecrosis of the jaw**			
Immunosuppressant medication	24.628	5.829, 104.053	0.000
Indication: other	6.168	1.464, 25.993	0.013
Surgical: with bone removal	6.086	1.449, 25.551	0.014
Antibiotics given: after	6.435	1.285, 32.232	0.024
**Oro‐antral communication**			
Surgical: with bone removal	4.264	1.144, 15.899	0.031
Clinician: oral surgeon or oral surgery registrar	4.150	1.312, 13.125	0.015
Anaesthesia: LA	0.277	0.088, 0.877	0.029
Anaesthesia: GA	3.621	1.145, 11.448	0.028

Use of general anaesthesia was associated with higher rates of nerve impairment (OR 9.49), trismus (OR 6.08) and oro‐antral communication (OR 3.62), whereas local anaesthesia was protective in many cases. Extractions performed by student clinicians were more likely to be associated with alveolar osteitis (OR 2.29) and abscess formation (OR 5.33). However, they were less likely to result in nerve impairment or trismus than specialists. Other risk factors included immunosuppressant use (OR 24.63 for osteonecrosis), impaction as an indication (associated with multiple complications) and use of preoperative rinses (linked to higher haemorrhage risk).

## 4 Discussion

The overall complication rate for extractions in this study was 11.0%, which falls within the range reported in the literature [39, 40, 41]. Regarding patient‐related factors, although older age has been associated with increased complications [42, 43], only trismus was significantly associated with the 65+ age group in this study. No significant gender‐related differences were observed, although females comprised 51.9% of the sample and accounted for 56% of complication cases, consistent with previous inconclusive findings [20, 44, 45, 46, 47]. While cardiovascular disease and blood thinning medications have been linked to increased post‐operative bleeding and cardiac events [48, 49, 50], this study did not screen for systemic complications; only long‐standing pain was associated with hypertensive patients. Smoking was significantly associated with alveolar osteitis, in agreement with reports showing up to a 300% increased risk [29]. Taking at least one medication was also associated with alveolar osteitis, though interpretation is limited due to heterogeneity in medication types. Immunosuppressants were associated with jaw osteonecrosis, which is well documented in the literature [51]. Uncontrolled diabetes was associated with alveolar osteitis and abscess formation, supporting prior findings linking hyperglycaemia to impaired healing [52, 53, 54], although this remains a topic of debate. Novel findings included associations between hypertension and long‐standing pain, epilepsy and haematoma, and allergies and trismus.

Tooth‐related findings were consistent with the literature. Although impaction angulation was not assessed, impacted teeth were associated with haematoma, facial cellulitis, trismus, nerve impairment and long‐standing pain, in line with previous research on impaction severity and trismus risk [14, 40, 55]. Surgical extractions, particularly those involving bone removal, were strongly associated with oro‐antral communication, nerve impairment, haematoma, osteonecrosis, trismus and long‐standing pain, echoing past studies on surgical complexity and infection risk [24]. Mandibular extractions were associated with a higher risk of nerve impairment, trismus and alveolar osteitis, reflecting established knowledge of increased risk with lower third molars [56]. Pericoronitis was significantly associated with long‐standing pain, in agreement with prior findings [24]. The ‘Indications: Other’ group was associated with jaw osteonecrosis, supporting links between MRONJ and pathologies such as root fractures and periapical disease [57].

Among clinician‐related factors, student clinicians were associated with the highest rate of post‐operative complications (10.86%), followed by general dentists (9.88%) and oral surgeons/oral surgery residents (6.39%). These findings are consistent with existing literature [24]. Despite this, student clinicians were linked with a decreased risk of nerve impairment and trismus, likely reflecting their simpler procedures; only 10.6% of their extractions were surgical, compared to 55.8% for general dentists and 75.8% for specialists. This is further influenced by institutional policy, which refers complex medical histories and surgical cases to oral surgery. Extractions under local anaesthesia were associated with an increased risk of alveolar osteitis, possibly due to vasoconstrictor‐related ischaemia [31, 58], although this association remains debated [59, 60, 61]. Extractions under general anaesthesia were associated with increased risks of haematoma, nerve injury, trismus and oro‐antral communication. Although the nerve impairment finding is well supported [62], the others require further validation.

Unexpected findings included the association of chlorhexidine mouth rinse with haemorrhage and abscess, which contradicts studies demonstrating its protective role against alveolar osteitis [33, 63, 64]. Variability in mouth rinse protocols and confounding factors may explain this. Antibiotic use was associated with an increased risk of osteonecrosis and nerve impairment, whereas previous literature has suggested a protective role [65]. A systematic review reported low‐certainty evidence that prophylactic antibiotics may reduce infection and alveolar osteitis in healthy individuals [66], though findings remain inconclusive. Antibiotic stewardship remains important, as indiscriminate use may not benefit all patients [67, 68]. Nevertheless, immunocompromised individuals or those with cardiac conditions may still benefit from targeted prophylaxis [69].

### 4.1 Limitations

As a cross‐sectional, retrospective study, causal relationships between risk factors and post‐operative complications cannot be established; only associations are observed. The reliance on clinical records introduced potential misclassification bias due to variable documentation quality or inaccurate patient‐reported information (e.g., medications, health status, smoking, post‐operative care). Survivor bias may have arisen from excluding records without updated medical histories or extraction indications, and attrition bias is likely, as post‐operative reviews are not routine at OHCWA, limiting the detection of complications that may have occurred after the initial appointment. Generalisability is also limited due to the specific demographics served by OHCWA. The patient cohort may differ from the broader population, particularly in age and socio‐economic status. Notably, 30% of the study population was aged ≥ 65 years, compared to only 13% of the general Western Australian population [70].

### 4.2 Clinical Implications to Future Research

Given the association between uncontrolled diabetes and increased risk of alveolar osteitis and abscess, delaying non‐urgent extractions until glycaemic control is achieved may reduce complications. Routine blood glucose checks at the start of appointments could support this approach. Hypertension was linked to long‐standing pain, though it is unclear whether this applies to all hypertensive patients or only those with poor control. If future research confirms the latter, preoperative blood pressure screening could help identify high‐risk patients and minimise post‐operative morbidity. Mandibular extractions were associated with higher rates of nerve impairment, trismus and alveolar osteitis. To mitigate these risks, clinicians could enhance post‐operative instructions with jaw exercises [71], and consider socket suturing to stabilise the clot and reduce the risk of alveolar osteitis [72, 73]. Finally, minimising surgical interventions where possible remains critical. Effective triage and ensuring appropriately skilled clinicians perform complex extractions may help reduce complication rates across patient groups [74].

## 5 Conclusion

In conclusion, although the individual complication prevalences were low, the overall prevalence of post‐extraction complications suggests that approximately 1 in 10 patients experience some form of complication. This research also highlights associated risk factors that clinicians can identify before the procedure, potentially reducing the likelihood of post‐extraction complications. Patients with uncontrolled diabetes and hypertension may benefit from better management of these conditions before undergoing dental extraction. Avoiding surgical extraction techniques where possible may further reduce the complication rates.

## Author Contributions

**Peter Dignam:** methodology, data curation, formal analysis, investigation, writing – review and editing, writing – original draft. **Mariam Elshafey:** data curation, investigation, writing – original draft, writing – review and editing. **Aparna Jeganathan:** investigation, data curation, writing – original draft, writing – review and editing. **Magdalen Foo:** supervision, writing – review and editing. **Joon Soo Park:** formal analysis, methodology, writing – original draft, writing – review and editing, supervision. **Manorika Ratnaweera:** conceptualization, writing – review and editing, supervision.

## Ethics Statement

The study was granted an exemption from ethics review by the Human Research Ethics Committee at the University of Western Australia (Exemption Number—2023/ET000651).

## Conflicts of Interest

The authors declare no conflicts of interest.

## Supporting information


Appendices S1–S2

## Data Availability

The data that support the findings of this study are available from the corresponding author upon reasonable request.
