# Supplementary material for: Prevalence and Risk Factors of Post‐Extraction Complications in a Western Australian Tertiary Dental Hospital: A Retrospective Cross‐Sectional Study
Source: Aust Dent J. 2025 Jun 10;70(4):266–74. doi: 10.1111/adj.13082 (PMC12661133; doi:10.1111/adj.13082)
Supplement: Supplementary file 1 — Appendices S1–S2 [file ADJ-70-266-s001.docx]

**Appendix S1 - Analysed variables and their prevalence**

## *Patient-related variables*

| **Category** | **Subcategories** | **Prevalence** |
| --- | --- | --- |
| **Age** | Numerical age | N/A |
|  | Age 65+ | N = 707 |
| **Gender** | Female | N = 1216 |
|  | Male | N = 1119 |
|  | Other | N = 8 |
| **Medication** | Any medications | N = 1429 |
|  | Immunosuppressants | N = 153 |
|  | Blood Thinner | N = 372 |
|  | Synthetic oestrogen/ Progesterone | N = 47 |
| **Smoking status** | Smoker | N = 866 |
|  | Smoker: Current | N = 554 |
|  | Smoker: Previous | N = 312 |
| **Health status** | No health conditions | N = 617 |
|  | Allergy | N = 488 |
|  | Surgical prosthesis | N = 122 |
|  | Low blood pressure | N = 60 |
|  | Normal blood pressure | N = 1583 |
|  | High blood pressure | N = 700 |
|  | Heart conditions | N = 304 |
|  | Bleeding conditions | N = 171 |
|  | Respiratory conditions | N = 433 |
|  | Epilepsy | N = 40 |
|  | Infectious disease | N = 81 |
|  | Diabetes mellitus | N = 276 |
|  | Diabetes mellitus: Controlled | N = 56 |
|  | Diabetes mellitus: Uncontrolled | N = 15 |
|  | Chemotherapy/ Radiotherapy | N = 209 |
|  | Chemotherapy/ Radiotherapy: Any head and neck radiotherapy | N = 117 |
|  | Chemotherapy/ Radiotherapy: No head and neck radiotherapy | N = 71 |
|  | Chemotherapy/ Radiotherapy: Chemotherapy only | N = 3 |
|  | Chemotherapy/ Radiotherapy: Type unknown | N = 18 |
|  | Depression/ Anxiety | N = 523 |
|  | Other mental health conditions | N = 237 |
|  | Recent hospitalisation | N = 126 |
|  | Pregnancy | N = 17 |

## *Tooth-related variables*

| **Category** | **Subcategories** | **Prevalence** |
| --- | --- | --- |
| **Indication For Extraction** | Hopeless periodontal prognosis | N = 215 |
|  | Hopeless restorative prognosis | N = 1023 |
|  | Impacted | N = 508 |
|  | Pericoronitis | N = 134 |
|  | Elective | N = 266 |
|  | Other | N = 210 |
| **Procedure Complexity** | Non-surgical: ADA Item code 311 | N = 1255 |
|  | Non-surgical: ADA Item code 314 | N = 48 |
|  | Surgical: ADA Item code 322 | N = 361 |
|  | Surgical: ADA Item code 323 | N = 172 |
|  | Surgical: ADA Item code 324 | N = 507 |
| **Dental Arch** | Maxillary | N = 1150 |
|  | Mandibular | N = 1193 |

## *Clinician-related variables*

| **Category** | **Subcategories** | **Prevalence** |
| --- | --- | --- |
| **Operator Experience** | Student clinician | N = 847 |
|  | General dentist | N = 901 |
|  | Oral surgeon or Oral surgery resident | N = 595 |
| **Anaesthesia and Conscious Sedation** | Local anaesthesia | N = 1684 |
|  | General anaesthesia | N = 657 |
|  | Conscious sedation (Inhalation/ Oral/ Intravenous) | N = 16 |
| **Mouthrinse** | Mouthrinse given/ Prescribed | N = 292 |
|  | Mouthrinse given/ Prescribed: Before | N = 264 |
|  | Mouthrinse given/ Prescribed: After | N = 64 |
| **Antibiotics** | Antibiotics prescribed | N = 154 |
|  | Antibiotics prescribed: Before | N = 28 |
|  | Antibiotics prescribed: After | N = 117 |
| **Gel Foam** | Gel Foam Placed | N = 331 |

## ADA - Australian Dental Association

**Appendix S2 - Terminology legends**

*Medical History terms and associated definitions*

| **Term** | **Definition** |
| --- | --- |
| **Former smoker** | Explicitly stated in the clinical notes that the patient used to smoke and is not currently smoking or the patient has quit smoking. |
| **Current smoker** | Explicitly stated in the clinical notes that the patient still smokes. |
| **Smoker** | Includes both current and former smokers |
| **Controlled diabetes** | Explicitly stated in the clinic notes or HbA1C recorded as less than 7.0 |
| **Uncontrolled diabetes** | Explicitly stated in the clinical notes or HbA1C recorded as more than 7.0 |
| **Head and Neck radiotherapy** | Any radiation to the base of skull, facial bones, sinuses, orbits, salivary glands, oral cavity, oropharynx, larynx, thyroid, facial and neck musculature and lymph nodes draining these areas |
| **No health conditions** | Patient reported no health conditions including pregnancy or any other conditions listed in the International Classification of Diseases |

*Indication for extraction terms and associated definitions*

| Term | Definition |
| --- | --- |
| **Hopeless periodontal** | Explicitly stated in the clinical notes or mention of severe attachment loss where extraction is necessitated |
| **Unrestorable** | Explicitly stated in the clinical notes or mention of not enough tooth structure to be able to restore the tooth |
| **Impaction** | Explicitly stated in the clinical notes |
| **Elective** | Explicitly stated in the clinical notes or mention of various options besides extraction proposed to the patient and the patient opting for extraction |
| **Pericoronitis** | Explicitly stated in the clinical notes |
| **Other** | Any other indications, such as cysts, tumours, non-functional, etc., |

*Clinician-related identifiers*

| **Factor** | **Identifiers** |
| --- | --- |
| **Mouthrinse** | Any mouthrinse given by the operating clinician to the patient either before or after the procedure including prescriptions written to the patient |
| **Antibiotics** | Any antibiotic prescribed to the patient either by the operating clinician or any health professional in relation to the dental extraction |
| **Gel foam** | Specifically mentioned gel foam, intra-socket haemostatic agent, or mentioned a brand of gel foam |

*Post-operative complication terms and associated definitions*

| **Term** | **Record screening guideline** |
| --- | --- |
| **Alveolar osteitis** | Explicitly stated in clinical notes, ‘dry socket’ terminology was used, exposed bone with no signs of infection, or mention of no blood clot present in the extraction site |
| **Osteomyelitis** | Explicitly stated in clinical notes or mention of infection of the bone or large bone sequestrum |
| **Abscess** | Explicitly stated in clinical notes or mention of localised collection of pus in relation to the extraction site |
| **Facial cellulitis** | Explicitly stated in clinical notes or mention of facial swelling post extraction alongside signs of systemic infection |
| **Long-standing pain** | Pain present beyond 7 days post-extraction. |
| **Haemorrhage** | Explicitly stated in clinical notes or mention of post extraction bleeding present for more than 8 hours |
| **Haematoma** | Explicitly stated in clinical notes or mention of extended duration of blood clotting |
| **Trismus** | Explicitly stated in clinical notes or mention of a period beyond 10 days of being unable to reach maximum mouth opening |
| **Oedema** | Explicitly stated in clinical notes or prolonged fluid retention in the extraction site beyond 7 days |
| **Inferior alveolar nerve damage** | Explicitly stated in clinical notes or mention of loss of sensation, hypoesthesia, hyperaesthesia, dysesthesia or paraesthesia in relation to the inferior alveolar nerve |
| **Osteonecrosis of the jaw** | Explicitly stated in clinical notes or mention of non-healing exposed, necrotic bone in patients who take anti-resorptive medication and/or have recent radiotherapy that extends to the extraction site |
| **Oral-antral communication** | Explicitly stated in clinical notes or mention of a communication between the extraction site and the maxillary sinus |
